# Gamified Digital Solutions for Tinnitus Health Literacy: The Erasmus+ Project TinWise

**DOI:** 10.1049/htl2.70072

**Published:** 2026-06-15

**Authors:** Evangelos Paraskevopoulos, Marios Avraamides, Panagiotis Bamidis, Christian Dobel, Christos I. Ioannou, Cosima Lukas, Birgit Mazurek, Andria Shimi, Efstathios Sidiropoulos, Kurt Steinmetzger, Eleftheria Vellidou

**Affiliations:** ^1^ Department of Psychology University of Cyprus Nicosia Cyprus; ^2^ Center for Applied Neuroscience University of Cyprus Nicosia Cyprus; ^3^ CYENS – Centre of Excellence Nicosia Cyprus; ^4^ Lab of Medical Physics and Digital Innovation School of Medicine Aristotle University of Thessaloniki Thessaloniki Greece; ^5^ Department of Otorhinolaryngology Jena University Hospital Friedrich Schiller University Jena Germany; ^6^ Department of Linguistics and Translation City University Hong Kong Hong Kong China; ^7^ Department of Rehabilitation Sciences Cyprus University of Technology Limassol Cyprus; ^8^ Tinnitus Center Charité – Universitätsmedizin Berlin Berlin Germany; ^9^ NGNC – NextGrowth Novelty Corporation Thessaloniki Greece; ^10^ Institute of Communication and Computer Systems (ICCS) Athens, GR Greece

**Keywords:** active hearing, bioacoustics, gamification, healthcare

## Abstract

Tinnitus is a common auditory symptom – sound perceived without an external source. Chronic tinnitus affects about 65 million adults in the EU, often impairing quality of life through distress, sleep problems, anxiety, and depression. Yet, management remains inconsistent across Europe due to patient heterogeneity and lack of standardised care. Health literacy is increasingly recognised as key to empowering patients in self‐management and reducing burden. The Erasmus+ project *TinWise* addresses this by creating a gamified health literacy platform for tinnitus patients and educating healthcare professionals on digital self‐help strategies. Building on *Tin‐TRAC*, which developed an open‐access e‐learning platform, *TinWise* integrates gamification to enhance motivation, engagement, and knowledge retention. The project applies the OPHELIA framework to optimise literacy strategies and follows the ASPIRE learning construct for co‐creating content. *TinWise* will deliver interactive, co‐created digital games to promote self‐help literacy, an AI‐powered chatbot for real‐time guidance, and a patient‐linking service to enhance community support. It also aims to train healthcare professionals using reusable learning objects, supporting the integration of digital tools into tinnitus care. Through modern technology and inclusive design, *TinWise* supports European priorities in digital education, patient empowerment, and healthcare innovation, ultimately improving tinnitus care and outcomes across Europe.

## Graphical abstract

TinWise is an Erasmus+ project that develops a gamified health literacy platform to support tinnitus patients in self‐management and educate healthcare professionals on digital care strategies. Building on the Tin‐TRAC e‐learning platform, it integrates interactive games, an AI chatbot, and community tools, following the OPHELIA and ASPIRE frameworks. The project aims to improve tinnitus care through digital innovation and inclusive design.

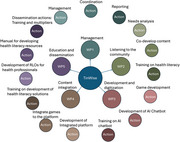

## 1 Background

Tinnitus is an auditory symptom characterised by the perception of sound in the absence of an external source. These phantom sounds, often described as ringing, buzzing, or hissing, can be intermittent or constant and vary in intensity [1]. While many individuals experience transient tinnitus at some point in their lives, chronic tinnitus persists beyond six months and may significantly impair daily functioning. Specifically, when the auditory sensation is associated with emotional and psychological distress, it can trigger a range of responses, including attention deficits, anxiety, sleep disturbances, irritability, and depression. In such cases, tinnitus is characterised as a disorder that may lead to social withdrawal and impaired occupational performance, contributing to a reduced quality of life [2]. Epidemiological data estimate that tinnitus affects between 10% and 30% of the adult population in the European Union (EU), with approximately 65 million adults experiencing this condition [3]. The economic impact is equally concerning: direct healthcare costs, productivity losses, and associated mental health treatments place a significant burden on healthcare systems across Europe. It is estimated that annual healthcare expenses for tinnitus patients range between EUR 500 and EUR 1500 per individual [4]. Given the high prevalence of tinnitus and its profound impact on both individuals and society, there is an urgent need for innovative, patient‐centred approaches to improve tinnitus management and reduce its strain.

The underlying causes of tinnitus remain a topic of ongoing investigation, with several physiological models proposed to explain the condition's onset and persistence. The most prevalent model suggests that tinnitus arises from maladaptive neural plasticity following auditory system damage, which leads to unbalanced inhibition/excitation activity in the auditory pathway [5]. But tinnitus is also related to consistent modifications in several intrinsic brain networks, including the default mode, auditory, dorsal attention, and visual resting‐state networks [6, 7]. However, tinnitus is not solely a physiological phenomenon; its chronification and the distress it causes are influenced by complex psychological and neurological factors. Various models attempt to explain how tinnitus transforms from a transient perception into a chronic condition accompanied by significant emotional distress: the recent triple network model points to dysfunctions in the interactions between the salience, default mode and central executive networks as key mechanisms [8]. Complicating matters further is the substantial heterogeneity among tinnitus patients. Differences in genetic profiles, auditory phenotypes, comorbidities, and psychological traits make it challenging to apply a one‐size‐fits‐all approach to treatment [1]. This variability extends to clinical practices across Europe, where tinnitus management strategies differ considerably between countries [9] and even between healthcare providers within the same region. Patients often experience fragmented care, receiving multiple referrals from one specialist to another without a clear, standardised treatment pathway [10]. This lack of coordination leaves many patients feeling unsupported in their journey to manage tinnitus, underscoring the urgent need for more cohesive, patient‐centred management strategies that address both the physiological and psychological dimensions of the condition.

It is important to note that tinnitus is not bothersome for all individuals [11]. While the presence of phantom sounds defines tinnitus as a symptom, it becomes a disorder only when these sounds are perceived as bothersome and interfere with daily life [2]. This distinction is critical in understanding the variability in tinnitus experiences among patients. Maybe the most prominent reason for this difference lies in the active nature of hearing as a sensory modality. Hearing is not a passive process; it involves complex cognitive functions, including attention, inhibition, and the ability to filter out irrelevant sounds from the auditory scene [12]. These processes play a crucial role in determining whether a sound – phantom, internal, or external – becomes part of one's conscious awareness and its perceived loudness [13]. Emotional responses, salience, and motivational factors further influence the direction of attention toward or away from certain sounds. In this context, the emotional characteristics of tinnitus, its perceived importance, and the individual's ability to shift attention away from it are key factors in whether tinnitus becomes a source of distress. The resulting capacity for compensation highlights the active role that patients can play in managing their condition [14]. However, for patients to take on an active role in self‐management, they must first have a comprehensive understanding of their condition and the factors that influence it. Health literacy, therefore, becomes a critical component in empowering patients to manage tinnitus more effectively and mitigate its impact on their quality of life.

Health literacy encompasses an individual's capacity to actively search for, comprehend, and apply health‐related information. The Council of Europe acknowledges that health literacy makes an important contribution to safeguarding the human rights principle of equitable access to healthcare and empowering citizens [15]. In this context, the patient is seen as an active participant rather than a passive recipient of healthcare. The European Health Literacy Study [16] showed that limited health literacy is a challenge in Europe and constitutes a problem not only for vulnerable groups but also for the general population. Currently, limited initiatives offer systematic approaches for health literacy, especially for chronic conditions, for which filtering condition‐related information and active engagement is important [17].

Despite the recognised importance of health literacy in tinnitus management, most existing resources remain text‐heavy, passive, or fragmented, offering limited support for sustained engagement or for translating knowledge into active self‐management. Tinnitus patients often report difficulty maintaining motivation and applying coping strategies in daily life, particularly when distress, attentional exhaustion, or inconsistent clinical guidance interfere with learning. Consequently, there is growing interest in interactive, patient‐centred approaches that can enhance engagement, support experiential learning, and promote the development of practical coping skills. Gamified health literacy tools offer a promising avenue by combining evidence‐based education with engaging, feedback‐rich experiences that foster active participation. The TinWise project builds on this rationale by developing gamified, co‐created resources designed to empower patients, improve self‐management, and complement the diversity of clinical practices across Europe.

## 2 Implementation

### 2.1 Towards a Co‐Creatively Developed Gamified Health Literacy Platform for Tinnitus

To address the pressing need for improved health literacy in tinnitus management, the Erasmus+ project Tin‐TRAC (https://tin‐trac.eu/) developed an open‐access e‐learning platform (MOOC) aimed at tinnitus researchers, clinicians, and patients. This MOOC is already developed and accessible under the following link: https://tin‐trac.med.auth.gr/moodle/. This platform provides a shared foundation of tinnitus‐related knowledge, fostering a common understanding across stakeholders. Building on this initiative, the follow‐up project TinWise (https://tinwise.eu/) introduces a gamified approach to patient education, aiming to enhance engagement, motivation, and interactivity in tinnitus self‐management. The gamified interventions of TinWise are yet to be developed. Gamification integrates elements such as challenges, rewards, and progress tracking into the learning process, transforming knowledge acquisition from a passive experience into an active and engaging one [18]. By incorporating these elements, the TinWise project seeks to bridge the gap between knowledge and action, empowering tinnitus patients to become proactive participants in managing their condition. Through gamified learning strategies, patients can better understand their symptoms, develop coping mechanisms, and train on implementing self‐help strategies in their daily lives. This approach represents a shift from traditional patient education to a dynamic, interactive model that leverages modern technology [19] to improve patient outcomes and promote self‐efficacy in tinnitus care.

TinWise is an Erasmus+ project, funded as a cooperation partnership in vocational education and training under the Grant Agreement number 2024‐1‐CY01‐KA220‐VET‐000252433. The project was officially started on 01 December 2024 and comprises six educational, research, and clinical centres and an SME in three European countries, namely Cyprus, Germany, and Greece. Tin‐TRAC's administration is located in Cyprus, where it is managed by project coordinator Evangelos Paraskevopoulos at the Department of Psychology in the University of Cyprus.

### 2.2 Concept and Methodology

TinWise addresses the key priorities outlined, focusing on the digital transformation through digital readiness, resilience, and capacity in chronic disorders. The proposed project has a dual goal of developing a health literacy platform for tinnitus patient use (as an example and blueprint for a chronic disorder) and teaching healthcare professionals the benefits of self‐help through digital means such as literacy platforms or health tracking applications. TinWise will exploit the OPHELIA framework [20] for optimising health literacy processes to develop the structure of a health literacy solution and enrich the approach with gamification and reusable learning object (RLO) principles. It will follow the ASPIRE learning construct [21, 22] (A – Aims, S – Storyboard, P – Population and Production, I – Integration, R – Release, and E – Evaluate) to co‐create the content of the platform, developing further the knowledge and tools gained from the Erasmus+ project TinTRAC (https://tin‐trac.eu/). Thereby, it will offer valuable information tailored to the needs of tinnitus patients and clinicians and translate knowledge into action via gamified interventions. Alongside this, TinWise will educate professionals on the benefits of self‐help and the soft skills on how to encourage it via digital means in chronic disorders, covering the corresponding pan‐European need.

Hence, TinWise follows a literature‐based, structured approach to achieving its objectives with tangible outcomes. The first objective is the development of a gamified health literacy platform for chronic disorders, using tinnitus as a case study. This platform aims to empower patients by transforming learning into an interactive experience, utilising gamification principles to enhance motivation and translate knowledge into action. A co‐creation approach will ensure inclusivity, with equal gender representation among patients contributing to the development of engaging and meaningful games. Implementation will involve collaboration with patient associations to design three interactive games that encourage self‐help literacy actions (described below). Additionally, an AI‐powered chatbot, trained on content from the TinTRAC Erasmus+ project, will facilitate discussions during gameplay, while an integrated service will connect willing patients locally via external communication channels. This objective aligns with European priorities in digital transformation, innovative learning spaces, and active patient engagement. Another objective focuses on educating healthcare professionals about the benefits of self‐help and equipping them with digital tools to support patient empowerment. This will be achieved through the development of RLOs that provide structured guidance on self‐help strategies. These RLOs will be integrated into the existing TinTRAC e‐learning platform, ensuring sustainability and broader applicability to other chronic conditions.

The implementation of TinWise's objectives will result in six key project outcomes (PO). These include a needs analysis and co‐creation report for game content (PO1), the development of gamified health literacy games (PO2), the creation of an AI chatbot and patient‐linking service (PO3), the establishment of the health literacy platform (PO4), the development and integration of RLOs for healthcare professionals (PO5), and a comprehensive manual on developing gamified health literacy resources for chronic disorders (PO6). Additionally, TinWise will facilitate six learning and teaching events tailored for both patients and healthcare professionals, alongside three dissemination events to maximise project impact. By addressing digital transformation, health literacy, and inclusivity, TinWise's objectives and activities are closely aligned with Erasmus+ priorities and broader European frameworks for digital education and healthcare innovation.

**FIGURE 1:**
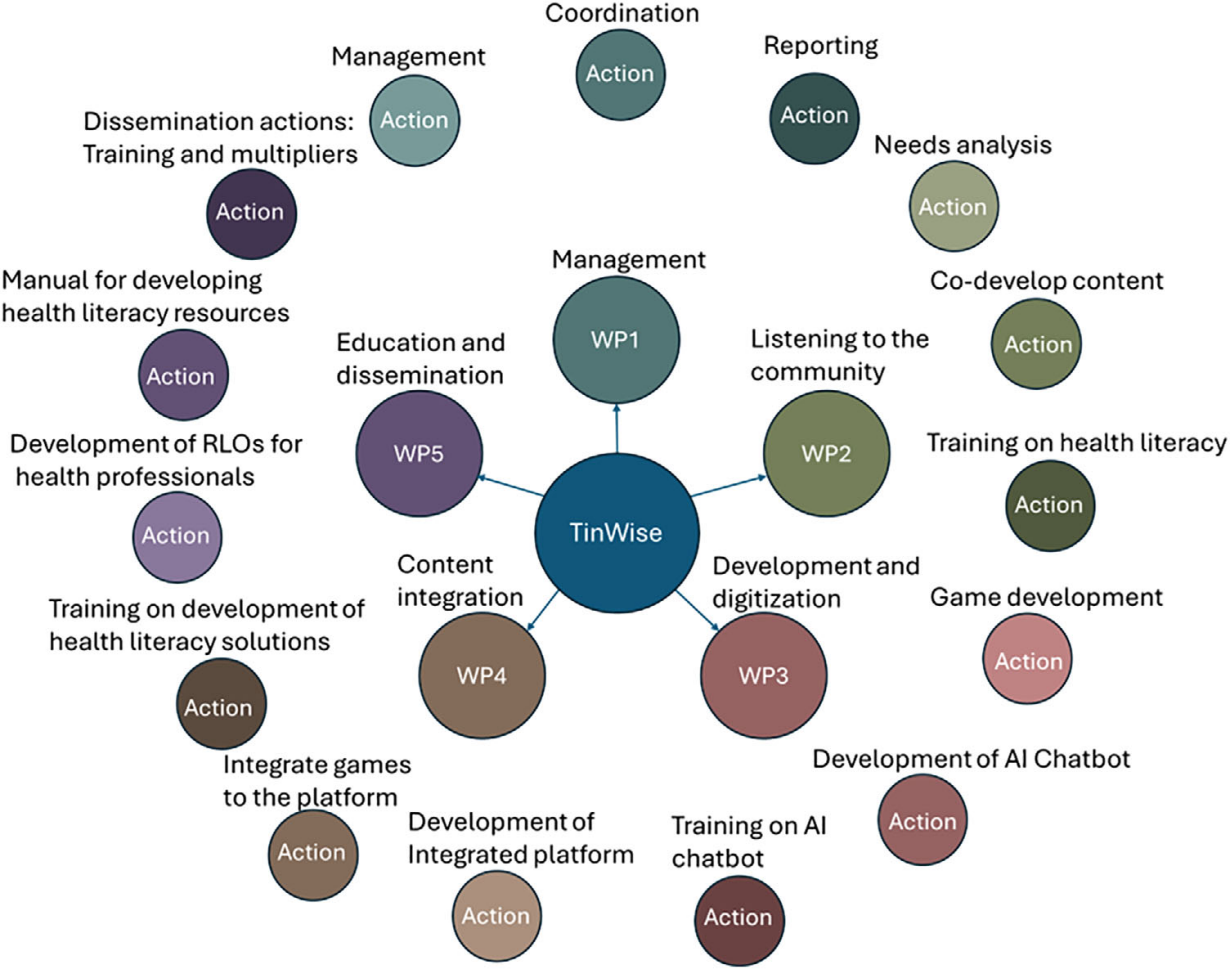
Overview of the TinWise project structure. The central node represents the TinWise platform, connected to the five work packages (WP1–WP5), each shown in a distinct colour. Surrounding nodes summarise the key actions within each work package, including coordination (WP1), needs analysis and co‐creation (WP2), game and chatbot development (WP3), platform integration (WP4), and professional education and dissemination (WP5). All abbreviations and colour codes are explained in the legend.

The successful implementation of TinWise's objectives and project results is structured around five distinct work packages (WPs), each focusing on a crucial aspect of the project: (a) WP1: Management and Coordination – This WP ensures effective project administration, reporting, and communication among partners to support timely delivery and quality assurance. (b) WP2: Listening to the Community – This WP involves conducting a needs analysis and co‐creating content with tinnitus patients and healthcare professionals. Through co‐creation events, TinWise will ensure that the developed resources align with real‐world patient needs and professional requirements. (c) WP3: Development and Digitisation – Building on the findings of WP2, this WP focuses on developing the health literacy platform's core content. It includes the design and digitisation of gamified learning resources, ensuring accessibility, inclusivity, and engagement. (d) WP4: Platform Integration and EU Linkages – This phase integrates all content into a unified health literacy framework, ensuring that the platform operates seamlessly. Additionally, WP4 will link TinWise with existing EU services and tinnitus‐related platforms, fostering synergies across European healthcare initiatives. and (e) WP5: Education and Dissemination – The final WP focuses on maximising TinWise's impact through training activities, dissemination events, and the creation of a practical manual for developing digital health literacy resources. This concept, depicted in Figure 1, ensures the sustainability and scalability of TinWise beyond the project's duration.

### 2.3 Listening to the Community: Needs Analysis and Co‐Creation of Content

This WP provides the empirical foundation for the TinWise project by engaging with the tinnitus patient community and facilitating knowledge exchange. Building on the methodologies and findings of the Erasmus+ TinTRAC project, the first objective (O1) involves conducting a web‐based survey to assess the tinnitus‐related educational needs of patients across Europe. The survey will expand on previous research [10] by focusing on how patients apply tinnitus‐related knowledge in daily life. This will yield critical insights into gaps in health literacy and self‐help strategies, ensuring that the platform addresses patient needs effectively.

The second objective (O2) emphasises both learning from and teaching patients. Insights from O1 will inform the development of three gamified learning objects, co‐created with tinnitus patients to ensure relevance and engagement. Co‐creation events will refine the content and structure of these games, incorporating patient feedback to balance difficulty and motivation. The second part of this objective focuses on empowering patients with the skills to navigate and utilise the gamified health literacy platform effectively. Training sessions will be conducted to enhance patient understanding of self‐help tools, fostering active engagement in managing their condition. These efforts align with current tinnitus guidelines emphasising the importance of educational and self‐help approaches [11, 23].

### 2.4 Development and Digitisation – Building on the Community's Needs

This WP focuses on transforming the insights gained from WP2 into tangible digital tools that enhance tinnitus health literacy. The primary objectives include developing three gamified learning experiences (O1), integrating an AI‐powered chatbot and a patient‐linking service (O2), and training health professionals in chatbot development for health literacy applications (O3). By expanding on the co‐created game concepts from WP2, WP3 ensures that the digital games cater to diverse user needs, varying by age, severity, and engagement levels. These games will foster health literacy through interactive learning, making tinnitus self‐management more accessible and engaging.

Integrated within the activities of this WP are the core concepts of the three planned gamified activities, which will be further shaped by stakeholders: (a) Game 1: Cocktail Party Challenge: This activity trains selective auditory attention by asking players to track a target voice among multiple AI‐generated stories. Intermittent multiple‐choice questions ensure active listening, and difficulty varies through narrative complexity and voice characteristics. Achievements reflect the highest difficulty level reached. (b) Game 2: Jungle Awareness: This game develops auditory localisation and awareness in a nature‐inspired environment. Players identify the spatial origin of animal sounds using rapid directional responses. Accuracy and reaction time combine into a unified achievement index. (c) Game 3: Spot the Musical Pattern: The final gamified activity strengthens auditory pattern recognition by prompting players to detect timbre, rhythm, or melody targets within musical excerpts. Levels progress from instrument recognition to rhythmic and melodic pattern tracking, with performance scored via accuracy–reaction time metrics. These descriptions serve as initial plans that will be adapted through co‐creation activities implemented within WP2 and will be in the following developed within WP3.Upon completion of each gamified session, the score will be fed to the chatbot, which in turn will initiate an interaction session, leveraging validated knowledge from the TinTRAC platform to provide personalised self‐help guidance, thereby addressing individual patient concerns in real time. Its multilingual capabilities will enable cross‐language communication, fostering a global tinnitus support network. The patient‐linking service will allow users to share personal information voluntarily, enabling direct interactions and peer support. Finally, WP3 will include training sessions to empower healthcare professionals in developing and implementing chatbots based on validated resources, ensuring they can leverage digital tools to enhance patient education. The core rationale is that training healthcare professionals in chatbot development ensures that clinical experts – not solely technical developers – shape the educational content, conversational design, and safety guidelines embedded in the chatbot. This enhances clinical accuracy, supports sustainable updating of health literacy resources, and equips professionals with skills to extend digital self‐help strategies beyond the TinWise platform. These initiatives collectively support the broader goals of TinWise by promoting digital transformation in tinnitus care.

### 2.5 Platform Integration and EU Linkages

The integration of digital tools and resources within a centralised health literacy platform marks a significant step toward improving tinnitus management across Europe. By developing a unified digital hub, WP4 ensures that tinnitus patients, healthcare professionals, patient associations, and EU institutions can access validated information, interactive learning experiences, and support networks in one place. The inclusion of gamified content, the patient‐linking service, and an AI‐powered chatbot enhances engagement by providing tailored, user‐friendly resources. Additionally, linking TinWise with the Tin‐TRAC platform strengthens the continuity of previous initiatives, creating a more comprehensive ecosystem for health literacy. A strong emphasis on inclusivity ensures that the platform adheres to WCAG guidelines [24], making it accessible to individuals with disabilities, while multilingual support and diverse content formats accommodate various learning preferences.

Beyond the technical development, WP4 prioritises the dissemination of knowledge among healthcare professionals, equipping them with the skills to integrate digital tools into their everyday practice. By embedding RLOs into both TinWise and Tin‐TRAC, professionals gain access to evidence‐based health literacy resources that support patient education and self‐management. Training sessions further promote digital competence, encouraging professionals to co‐create and implement personalised solutions for chronic conditions. This initiative not only fosters a collaborative approach to tinnitus care but also contributes to a broader shift in healthcare, where digital literacy and innovative learning spaces become essential components of patient empowerment and engagement.

### 2.6 Education and Dissemination

The success of TinWise depends not only on developing innovative digital health literacy tools but also on ensuring that these resources are widely adopted and effectively utilised by healthcare professionals and patients. WP5 plays a crucial role in this process by implementing comprehensive education and dissemination strategies that enhance professional capacities, introduce innovative teaching methods, and maximise the project's impact. Through specialised training events, WP5 equips clinicians with the skills needed to integrate health literacy tools into patient care, fostering a patient‐centric approach to managing chronic disorders. The development of RLOs and a structured manual further supports this objective, providing professionals with accessible, evidence‐based resources that promote self‐help strategies through digital means. By bridging TinWise with the Tin‐TRAC platform, WP5 also ensures continuity with previous initiatives, strengthening the overall knowledge base and expanding opportunities for professional development.

In addition to training, dissemination activities are central to WP5, ensuring that TinWise reaches a broad audience across Europe. Multiplier events and webinars serve as key platforms for engaging stakeholders, including patient associations, clinicians, and researchers, fostering collaboration and encouraging the integration of health literacy principles into everyday clinical practice. These hybrid events, held across multiple countries, leverage digital technology to maximise outreach while maintaining interactive engagement. The structured approach to dissemination ensures that the project's findings and innovations contribute to ongoing advancements in digital health literacy, influencing both policy and practice. Ultimately, WP5 establishes a sustainable framework for education and engagement, reinforcing TinWise's long‐term impact on improving health literacy and patient empowerment in chronic disorder management.

## 3 Conclusions

The TinWise project represents a pivotal advancement in tinnitus management by bridging the gap between health literacy, digital engagement, and patient empowerment. By integrating gamification into health education, TinWise transforms traditional learning into an interactive experience that fosters self‐efficacy and active participation in tinnitus care. Through co‐creation with patients and healthcare professionals, the project ensures that its tools are both relevant and effective, addressing real‐world challenges in tinnitus management. Additionally, by linking with existing European initiatives such as Tin‐TRAC and leveraging digital solutions like AI‐powered chatbots and patient‐linking services, TinWise contributes to a broader shift towards accessible, patient‐centred care. Beyond tinnitus, the structured methodologies and reusable learning resources developed within TinWise offer a scalable model for other chronic conditions, emphasising the critical role of digital literacy in modern healthcare. As the project unfolds, its impact will extend beyond the immediate patient population, fostering a more inclusive and innovative approach to chronic disease management across Europe.

## Author Contributions

E.P. wrote the main manuscript text and prepared Figure 1. All authors (M.A., P.B., C.D., C.I.I., C.L., B.M., A.S., E.S., K.S., and E.V.) revised and reviewed the manuscript.

## Funding

TinWise is a Joint Project funded by the European Commission within the ERASMUS+ 2024 Programme, Key Action 2 – KA220‐VET – Cooperation partnerships in vocational education and training under the Grant Agreement Number 2024‐1‐CY01‐KA220‐VET‐000252433.

## Conflicts of Interest

The authors declare no conflicts of interest.

## Data Availability

Data generated through the project will be openly available.
